# Probiotics for Oral Candidiasis: Critical Appraisal of the Evidence and a Path Forward

**DOI:** 10.3389/froh.2022.880746

**Published:** 2022-04-14

**Authors:** Linda S. Archambault, Anna Dongari-Bagtzoglou

**Affiliations:** ^1^Department of Craniofacial Sciences, University of Connecticut Health Center, Farmington, CT, United States; ^2^Center for Quantitative Medicine, University of Connecticut Health Center, Farmington, CT, United States

**Keywords:** *Candida*, oropharyngeal candidiasis, probiotics, *Lactobacillus*, clinical trials, preclinical models

## Abstract

Oropharyngeal Candidiasis (OPC) is a mucosal fungal infection that is prevalent among patients with compromised immunity. The success of probiotics in treating chronic diseases with a microbial etiology component at other mucosal sites (i.e., gastro-intestinal, genitourinary and alveolar mucosae) has inspired research into the use of probiotics in the treatment of OPC. A growing body of research *in vitro* and in animal models indicates that some probiotic species and strains have inhibitory activities against *Candida albicans* growth, morphological switching, and biofilm formation. However, recent review and meta-analysis studies reveal a dearth of human randomized, controlled clinical trials on the efficacy of probiotics to treat or prevent OPC, while the majority of these have not based their selection of probiotic strains or the type of administration on sound pre-clinical evidence. In this mini-review, we assess the state of the field, outline some of the difficulties in translating lab results to clinical efficacy, and make recommendations for future research needed in order to move the field forward.

## Introduction

Oropharyngeal Candidiasis (OPC) is a common fungal infection affecting populations with immature or weakened immune systems including neonates, the elderly, AIDS patients, and those taking immunosuppressive drugs [1–4]. Like other diseases in which commensal microbes become pathogenic, OPC is associated with a dysbiotic state, an alteration in the composition or abundance of the oral health-associated host microbiota, contributing to a permissive environment for *Candida* spp. infection [5, 6]. An aging population, the rise in the use of immune-compromising treatments and the increasing prevalence of drug-resistant *Candida* strains make it necessary to find alternative treatments for OPC.

Probiotics are defined by the FAO/WHO as “live microorganisms which, when consumed in appropriate amounts in food, confer a health benefit on the host” [7]. Their use is believed to discourage pathogenic microbes and help the host microbiota to return to a health-associated, balanced state after disruptions [8]. Research has provided evidence supporting a positive role of some probiotic organisms in promoting gut, oral and vaginal health, but clinical studies showing no effect of probiotics also exist [9–11].

In this mini-review we explore the current evidence for the role of probiotics in the prevention and treatment of OPC. We will not summarize all work in this area, a task well-accomplished by recent systematic reviews [9, 12–14]. Rather, our intention is to critically appraise the state of the science on this topic by integrating the information gleaned from studies *in vitro* and in animal models with the results of human clinical trials, where possible. We discuss the pitfalls of each type of research and the difficulties encountered in translating results from laboratory research to benefits for patients, then suggest areas that need more research attention in the future.

## What Can We Learn from Clinical Trials?

Three recent systematic review and meta-analysis studies explored probiotics as treatment or prevention for OPC [15–17]. These studies restricted their analyses to randomized, controlled clinical trials (RCTs) which measured *Candida* burdens in colony-forming units (CFUs) from saliva samples or swabs of oral mucosal tissues. The meta-analyses included between 3 [18–20] and 8 clinical trials [21–25] that compared a probiotic to a control or placebo. All three meta-analyses found significant reduction in *Candida* counts in the oral environment as a result of probiotic treatment, although this did not always lead to significant clinical improvements. This suggests that the development of probiotic approaches holds promise in changing current clinical guidelines of OPC prevention or treatment, especially for immunocompromised patients and for patients with refractory or recurring infections.

### Bacterial Species Tested as Probiotics in Clinical Trials and Basis for Their Selection

Ideally, when probiotic strains are chosen for clinical trials involving OPC, it would be on the basis of experimental results, either *in vitro*, or in animal models, or both, that demonstrate their activity against *Candida* species. Typically, research begins with *in vitro* studies on the interactions between probiotic species and *Candida* species, proceeds to *in vivo* animal model studies to examine aspects of the interaction between probiotics, *Candida*, and the host, and then moves on to human clinical trials of the probiotic species that show promise. However, probiotics in fermented foods have been part of the human diet since ancient times and scientific study into the clinical benefits of probiotic bacteria for human health began in the late 19^th^ century when scientific methods of inquiry were not well-developed [26]. In addition, much of the early work in the study of probiotics to treat or prevent disease has focused on gastro-intestinal conditions [27]. Because of their presumed safety and benefits in intestinal mucosal diseases, some probiotic species have been tested in humans for their ability to prevent OPC without any prior experimental evidence supporting their anti-*Candida* activities [18, 22].

The justification behind the choice of probiotic species and strains was specified in 6 of the 8 RCTs covered by the 3 meta-analyses. Commercial probiotic preparations usually contain several species of bacteria, thus clinical trials often use a mixture of species or strains. *Lactobacillus* species were included in all but one of the RCTs. *L. rhamnosus* was the most commonly included species (4 studies) and *L. reuteri, L. acidophilus*, and *L. bulgaricus* were each used in 2 studies. Additional species in probiotic mixtures included *Bifidobacterium longum, Bifidobacterium bifidum, Propionibacterium freadenreichii*, and *Streptococcus thermophilus*. In two of the studies, no reason was provided for the choice of probiotic species or strain [22, 24]. Two of the RCTs used a mixture of 2 strains of *L. reuteri* [20, 25] citing this species' production of the antibacterial toxin reuterin and its ability to inhibit the *in vitro* growth of many *Candida* spp. [28]. *L. rhamnosus* Lr32 and *L. acidophilus* NCFM, which were compared in Miyazima et al. [23], had been previously found to reduce oral *C. albicans* numbers in an immunocompromised mouse model [29] and to disrupt *C. albicans* biofilms *in vitro* [30]. This same group conducted a RCT of a commercial, three-species mixture of *L. rhamnosus* HS111, *L. acidophilus* HS 101 and *Bifidobacterium bifidum* [19], citing studies that tested these and other *Lactobacillus* species against *Candida* growth *in vitro* [31, 32]. In one study intended to improve dental caries incidence in children, *Streptococcus salivarius* was chosen for its antagonism with *S. mutans* [21]; changes in both *Candida* and *S. mutans* numbers were recorded because of the known synergistic roles of these 2 organisms in dental caries [33, 34]. However, in this study, there was no significant difference in *Candida* oral burdens between probiotic treatment and placebo. To explain their choice of probiotic species, Hatakka and colleagues [18] cited a successful clinical trial which measured gastrointestinal colonization of *Candida* in neonates given *L. rhamnosus* GG [35] and a mouse study which found that probiotic treatment with either *Propionibacterium* JS or *L. rhamnosus* GG increased the proliferation of T- and B-cells [36]. The third strain in their study, *L. rhamnosus* LC705, was used in combination with *Propionibacterium* JS, which is a preservative against yeast in food manufacture [18]. Thus, it appears that justification for the use of specific probiotic strains in existing RCTs is variable, and the majority of these studies were not based on strong experimental *in vitro* evidence or on results with preclinical animal models of oral infection.

Few research groups have progressively investigated the same probiotic species *in vitro*, in an animal model, and in human clinical trials. One such group first found each of 2 *Lactobacillus* strains, *L. rhamnosus* LR32 and *L. acidophilus* NCFM, to be effective against OPC in an immunocompromised mouse model [29]. They then showed the ability of the same strains to interfere with adhesion and hyphal growth in the early stages of *C. albicans* biofilm formation *in vitro* [30]. In the ensuing human clinical trial, both strains reduced the oral *Candida* levels in denture wearers, one (LR32) performing even better than the positive control drug Nystatin [23]. The same group tested a commercial product containing different strains of the same two species, *L. rhamnosus* HS111 and *L. acidophilus* HS 101, in a successful clinical trial in denture-wearers [19]. These results however were not reproduced independently by a different group and have not led to widespread implementation of these probiotics in clinical practice.

*Lactobacillus rhamnosus* GG (LrGG) was patented for use as a probiotic in 1989 and there is an extensive body of clinical research justifying claims of its health benefits, mostly in the treatment of gastro-intestinal disorders [37, 38]. Many of the characteristics that make it promising as a probiotic for gut ailments could apply to the oral environment–it adheres to epithelial cells, promotes type 1 immune response, reduces inflammation by inhibiting macrophage activation and increases production of IL-10, IL-12 and TNFα in innate immune cells [37]. A great deal of *in vitro* and *in vivo* laboratory work has delved into the mechanisms behind this strain's ability to inhibit potentially pathogenic organisms directly and through its influence on host immune responses [37]. LrGG inhibits *C. albicans* adhesion and morphological changes [39], induces metabolic reprogramming by nutrient limitation [40], and may break down cell-wall components [41]. LrGG also reduces oral *Candida* counts in immunocompromised mice [42]. Although there is strong experimental and preclinical evidence supporting the potential efficacy of this strain in limiting OPC, it was used in only one of the above listed RCTs and then, only in combination with other species [18]. More RCTs should provide independent support for the benefits of this probiotic strain in the context of OPC treatment or prevention in humans.

### Choosing the Best Probiotics for the Oral Environment

While these clinical and laboratory studies are good first steps, it is evident that in order to make progress in the development of probiotics-based new therapies or prevention strategies for patients who are most susceptible to OPC, we will need to approach scientific inquiry in a more systematic way. This process should include more *in vitro* work to screen potential probiotics for their activity against *Candida* species and examine mechanisms of anti-fungal activity. Some criteria for a good probiotic for the treatment of intestinal illness (i.e., the ability to withstand the acid environment of the stomach, be metabolically active under the low oxygen tension in the gut, or to attach to intestinal epithelial cells) may not apply to the oral environment. In choosing the best probiotics for the oral environment, *in vitro* experiments are needed to explore attachment to oral epithelial cells and the ability to colonize the oral mucosa. It is also important to isolate, identify and test metabolites produced by effective species as these postbiotics could prevent OPC without the risks associated with introducing live organisms in immunocompromised patients who make up most of the susceptible populations [43]. Animal models that recapitulate the different host susceptibility states (i.e., immunocompromised, elderly, cancer patients) will be needed to discover the best probiotic species for each condition and also to test a variety of delivery methods.

## Pitfalls in Translating Laboratory Findings to Clinical Trials

Experimental work has provided evidence of several anti-*Candida* activities of *Lactobacillus* spp. that negatively affect *C. albicans* virulence characteristics including growth, yeast to hyphae transition, adhesion, and biofilm development [44, 45]. Antifungal mechanisms include production of acids, hydrogen peroxide, bacteriocins, and biosurfactants, as well as competition for adhesion sites. Probiotics can also activate protective functions of the immune system, affecting cytokine profiles, innate and adaptive immune responses, and *Candida* recognition by epithelial and immune cells [44, 45]. However, which of these protective mechanisms are active in the human oral environment is not fully understood. In the oral environment some of the probiotic protective functions may be compromised by dietary habits and nutrient availability, antagonistic interactions with a complex resident microbiota, and the salivary flow which may limit colonization and persistence.

With respect to persistence in the oral cavity, the impact of probiotic consumption on the oral microbiome is relatively unexplored. The question of whether probiotic organisms become stably incorporated into the human host oral microbiome is not often addressed in clinical trials. However, there is some evidence that while ingested organisms are often detected in the oral cavity of participants during probiotic treatment, they do not persist once treatment ceases [24, 46]. Nevertheless, because OPC is precipitated by an underlying dysbiosis in the oral microbiome [5, 6], it would be important to demonstrate that probiotics can encourage the recovery of a “healthy” microbiome in the oral environment after disruptions caused by antibiotics, cancer treatment or chronic disease, as they appear to do in the gut [8]. These important questions are beginning to be explored using next generation sequencing technology [47, 48] and require more investigation using preclinical models of infection.

It is clear that complex interactions between the fungus, the resident microbiota and the host immune system determine whether *C. albicans* remains commensal or becomes pathogenic in the oral environment [49, 50]. T helper-17 (Th17) cell-mediated immunity is crucial in maintaining commensal colonization of the oral mucosa by *C. albicans* [51, 52]. The host aryl hydrocarbon receptor (AhR) binds ligands that are products of tryptophan catabolism and activates IL-17/IL-22 production by Th17 cells. Lactobacilli metabolize tryptophan to produce an AhR ligand, indole-3-aldehyde, which triggers IL-22 production to protect from *C. albicans* infection and dampen inflammation [53]. Further exploration in this area is needed to reveal how probiotic organisms contribute to metabolic control of protective immune responses in the oral environment.

The effects of probiotic organisms on epithelial barrier function and host immunity have been extensively studied in the context of gastro-intestinal diseases [11, 54]. Probiotics affect host immunity at both the local and systemic levels. Systemic immune effects of probiotic consumption could influence the host's ability to control *Candida* in the oral environment without the requirement for probiotic organisms to take up residence there. For example, consumption of *Lactobacillus* strains in mice was tied to improvement in the function of both peritoneal and alveolar macrophages and protection against both lung and peritoneal infection by *C. albicans* [55]. These distant protective effects may extend to the oral environment.

Experimental evidence suggests that probiotic organisms prime epithelial and immune cells to respond differently to *Candida* challenge. Pretreatment with *Lactobacillus* strains before exposure to *C. albicans* causes changes in cytokine production and pattern recognition receptor expression in macrophages [56]. In epithelial (HeLa) cell lines, pretreatment with *L. crispatus* altered β-defensin and cytokine production and toll-like receptor expression in response to *C. albicans* [57]. To shed light on the role of probiotics in the oral environment, more work must be done using oral epithelial cell lines, primary epithelial cells and mucosal organotypic cultures [58]. It will also be important to differentiate between direct effects of the probiotic organisms on epithelial tissues vs. effects secondary to *Candida* growth and morphology, which can also alter immune responses [59].

## The Path Forward: Developing Better-Targeted Probiotics-Based Approaches

Evidence from RCT meta-analyses suggests that probiotic treatments may work better in preventing OPC, compared to treating established infection in susceptible patient categories [15–17]. This may indicate that short-term administration of probiotics cannot lead to changes in the oral microbiota that are sufficient in magnitude or duration to treat this infection. Interestingly, in these meta-analyses when studies that involved elderly patients or denture wearers were considered separately [18–20, 23], a stronger beneficial effect was found compared to including all studies and all susceptible patient groups together [15, 16]. A recent RCT, not included in the meta-analyses, confirmed this trend [60]. In other patient populations (children and adults), oral *Candida* numbers showed low or no responsiveness to probiotic treatments [21, 24, 25]. This evidence suggests that probiotics must be better targeted to specific vulnerable populations such as patients who are HIV positive, undergoing cancer treatment, or taking immunosuppressive drugs. *In vivo* animal models mimicking these conditions may be useful in developing a better-targeted, host immune susceptibility type-combined with a specific probiotic strain approach in treatment or prevention.

In the 8 RCTs mentioned above, the dose, timing, duration and delivery methods for probiotics were varied. Dosages in adult populations ranged from 7.2 x 10^7^ to 2 x 10^10^ CFUs per day. To put these numbers in perspective, a dosage of 10^10^ CFU per day was required to colonize the digestive tract and to treat acute gastroenteritis in children [61, 62], suggesting that better outcomes might have been achieved with higher doses in OPC studies with adults. Delivery methods included lozenges, cheese, yogurt, milk and the application of probiotic organisms directly onto denture surfaces. The timing of delivery ranged from once per day to 3 x per day and the duration of treatment ranged from 4 weeks to 3 months. No pilot studies were conducted to compare the efficacy of particular dosages, timing, delivery or duration and there is no indication of whether any of these variables are essential for success. Again, preclinical animal models could provide useful insights on these variables.

## Discussion

Based on recent studies, it is clear that relatively few clinical trials of probiotics for OPC were based on sound *in vitro* experimental data and/or appropriate preclinical animal models of infection. Because of the beneficial immunomodulatory effects of several probiotic strains, we suggest that further research is needed in animal models that recapitulate the specific host immune deficiency, to better target the probiotics to the specific underlying immune-compromising condition of susceptible patients.

It will also be important to further study the mechanisms of inhibition of *Candida* spp. by probiotic organisms and particularly to determine whether these mechanisms are active in the oral environment. Animal models should be used to determine the best methods for delivery of probiotics to the oral cavity before being applied in humans. Molecules with anti-*Candida* activity that are produced by probiotic species must be identified and their activity tested in appropriate animal models. Further, it will be essential to explore immune effects of probiotics both at the local level of the oral mucosa and at the systemic level and to determine how these changes in immune responses impact the susceptibility or severity of OPC.

## Author Contributions

LA researched the literature and wrote and edited the article. AD-B provided guidance and focus and edited the article. Both authors contributed to the article and approved the submitted version.

## Funding

This study was funded by NIH/NIDCR Grant RO1 DE013986 and NIH/NIGMS Grant RO1 GM127909.

## Conflict of Interest

The authors declare that the research was conducted in the absence of any commercial or financial relationships that could be construed as a potential conflict of interest.

## Publisher's Note

All claims expressed in this article are solely those of the authors and do not necessarily represent those of their affiliated organizations, or those of the publisher, the editors and the reviewers. Any product that may be evaluated in this article, or claim that may be made by its manufacturer, is not guaranteed or endorsed by the publisher.
